# Assessment of demineralized tooth lesions using optical coherence tomography and other state-of-the-art technologies: a review

**DOI:** 10.1186/s12938-022-01055-x

**Published:** 2022-12-03

**Authors:** Fatin Najwa Mohamad Saberi, Prema Sukumaran, Ngie Min Ung, Yih Miin Liew

**Affiliations:** 1 Department of Biomedical Engineering, Faculty of Engineering, Universiti Malaya, Kuala Lumpur, Malaysia; 2 Faculty of Dentistry, Oral & Craniofacial Sciences, King’s College London, London, England; 3 Clinical Oncology Unit, Faculty of Medicine, Universiti Malaya, Kuala Lumpur, Malaysia

**Keywords:** Optical coherence tomography, Caries, Tooth demineralization, Morphological changes, Optical properties, Tooth lesion

## Abstract

Tooth demineralization is one of the most common intraoral diseases, encompassing (1) caries caused by acid-producing bacteria and (2) erosion induced by acid of non-bacterial origin from intrinsic sources (e.g. stomach acid reflux) and extrinsic sources (e.g. carbonated drinks). Current clinical assessment based on visual-tactile examination and standardized scoring systems is insufficient for early detection. A combination of clinical examination and technology is therefore increasingly adapted. This paper reviews various procedures and technologies that have been invented to diagnose and assess the severity of tooth demineralization, with focus on optical coherence tomography (OCT). As a micron-resolution non-invasive 3D imaging modality, variants of OCT are now available, offering many advantages under different working principles for detailed analytical assessment of tooth demineralization. The roles, capabilities and impact of OCT against other state-of-the-art technologies in both clinical and research settings are described. (139 words).

## Introduction

Demineralization of tooth structure is an oral health problem that affects a large portion of the population regardless of age [1]. Demineralization can manifest as caries lesions or erosive lesions, leading to tooth ache, inflammation of the dental pulp and, eventually, tooth loss if left untreated. According to the 2017 Global Burden of Disease Study, tooth caries has high prevalence worldwide, affecting the permanent teeth of 2.3 billion adults and the primary teeth of more than 530 million children [1]. Apart from compromised aesthetics, dentinal hypersensitivity and reduced chewing functionality gave a great impact to a person’s daily living. Complicated treatment and high treatment costs are usually required in advanced cases.

Demineralization of dental hard tissue is preventable and very treatable in preliminary stages and the management is less complicated. In a clinical setting, both caries and erosive lesions are usually diagnosed through visual assessment and radiography, but these methods have low sensitivity in distinguishing the early stages of lesions [2, 3]. Therefore, various tools, such as tomographic imaging, have been introduced over the past two decades to improve the assessment of tooth lesions. Optical coherence tomography (OCT) is an optical imaging modality that is capable of performing non-invasive tomographic imaging at micrometre resolution. After its first introduction in 1991 by Professor James G. Fujimoto of the Massachusetts Institute of Technology in the United States, its usage took more than a decade to gain the approval of dentists and health authorities worldwide. The ability of OCT to detect subtle changes in the optical and morphological properties of tooth surface without the use of ionizing radiation provides an extra safety advantage for patients and clinicians. The use of OCT in combination with various image processing techniques has been actively explored and may further assist dental practitioners in diagnosing demineralized lesions in teeth objectively.

This review provides the details of different types of tooth lesions, clinical scoring systems and various imaging modalities for assessing demineralized lesions, with specific focus on OCT. The principles of OCT imaging and setups for dental applications are reviewed and discussed, with key quantification metrics for demineralization assessment.

## Clinical presentation of demineralized teeth and assessment tools (clinical and research)

### Aetiology and clinical presentation

Demineralization can be categorized into two groups based on aetiology. First group is caries lesions, which is caused by mouth acids produced by the proliferation of cariogenic bacteria during metabolism of fermentable carbohydrates. In the early stage, caries develops as a subsurface lesion—a chalky white appearance on an intact surface. As demineralization progresses, the tooth surface becomes eroded and a cavity forms.

The second group is known as erosive lesions, which is initiated from the chemical processes that dissolute the enamel and dentine by acids that are not produced from bacteria [4]. The aetiology includes consuming a large amount of acidic food and beverage, frequent vomiting due to gastroesophageal reflux disease (GERD), effects of certain medications, like anti-histamines and tranquilizers and salivary gland disorders (which results in low buffering capacity and flow rate) [5–9]. Erosive lesions have different morphologies and physical appearances than caries. Advanced erosive lesions will result in teeth discolouration, cupping out of occlusal surfaces leading to exposed dentine and broad concave lesions on enamel and dentine surfaces [10]. In the anterior teeth, these lesions may present with increased incisal translucency, incisal thinning and chipping and cupping out of the incisal edges [11]. Erosive lesions may also appear as shiny wear facets on tooth structure or restorations [6].

### Lesion scoring systems and challenges

Clinical diagnosis of caries and the assessment of its severity are mainly based on visual inspection using a standardized lesion scoring system. The two most commonly used scoring systems are the International Caries Detection and Assessment System (ICDAS) and Nyvad Criteria [12, 13]. Both scoring systems differ in terms of the criteria to determine severity level and tools used. ICDAS criteria were introduced in 2002 by the Swiss-based Fédération Dentaire Internationale (FDI), with score codes ranging from 0 to 6 (Table 1), to diagnose caries lesions based on surface characteristics and presence of sealants or restorations on the lesion area [12]. The Nyvad criteria (Table 2), by contrast, were introduced in 1999 to classify caries from a visual-tactile examination to achieve evidence-based management in clinical practice [13].

**Table 1:** ICDAS scoring system for visual assessment of caries lesion (Adapted from [12, 14, 15])

Code	Criteria
0	Represents sound tooth. No evidence of caries or slight change on enamel translucency after 5 s of drying
1	First visual changes in enamel after prolonged air drying. Opacity or discolouration, such as white or brown spot, may be visible at the edge of fissure or pit
2	Obvious visual changes in enamel at wet condition and visible lesion seen when dry
3	Localized enamel breakdown without visual changes of dentine involvement is seen during wet and after prolonged drying
4	Underlying dark shadow from dentine which is more easily seen when wet
5	Obvious caries and visible dentine are seen
6	Extensive distinct caries (more than half of the surface) with visible dentine

**Table 2:** Nyvad criteria scoring system for visual assessment of primary and secondary caries lesions (Adopted from [13])

Scores	Criteria
0	Sound teeth with normal translucency and texture of enamel, allowing slight staining
1	Presence of active caries on surface of enamel which appear as yellowish or whitish opaque spot. Feels rough when tip of probe is gently moved on top of the surface. Lesion may be extending along walls of fissure
2	Almost same morphology as score 1 (active caries is detected in enamel only). No softened floor is detected
3	Enamel and dentine cavity is easily visible to naked eye (inactive caries). Surface is soft or leathery when tip of probe is moved across the spot. Pulpal may or may not be involved
4	Inactive caries with intact surface. Enamel may appear as shiny or whitish, brownish or black spot. Surface of enamel feel hard and smooth when touch using tip of probe. Lesion is extending along fissure’s walls
5	Inactive caries with localized surface of microcavity on enamel only. No softened floor or undermined enamel detected with explorer
6	Inactive caries where shiny surface of cavity on enamel and dentine can be seen easily with the naked eye. Surface feels hard on gentle probing. No involvement of pulpal
7	Filling with sound tooth surface
8	Filling with active caries lesion that may be cavitated or non-cavitated
9	Filling with inactive caries lesion that may be cavitated or non-cavitated

Erosive lesions, on the other hand, are commonly graded using Basic Erosive Wear Examination (BEWE) that was introduced in 2008 by Bartlett et al*.* [7], whereby the lesions are classified into four levels of severity for three different parts of teeth—facial, occlusal and palatal surfaces (Table 3) [5]. BEWE is performed by assessing the physical appearance of teeth, such as the surface texture and percentage of hard tissue loss.

**Table 3:** BEWE scoring system to determine severity of erosive lesion (Adopted from [7])

Scores	Criteria
0	No erosion detected. Smooth and silky-shiny appearance with the absence of developmental ridges
1	Intact enamel is found cervical to lesion. Loss of surface enamel is detected. Width of concavity in enamel is wider than its depth is distinguished from toothbrush abrasion (wide and shallow concavity)
2	Dentine is involved for less than half of the tooth surface
3	Severe erosion with involvement of dentine more than half of the tooth surface

**Fig. 1:**
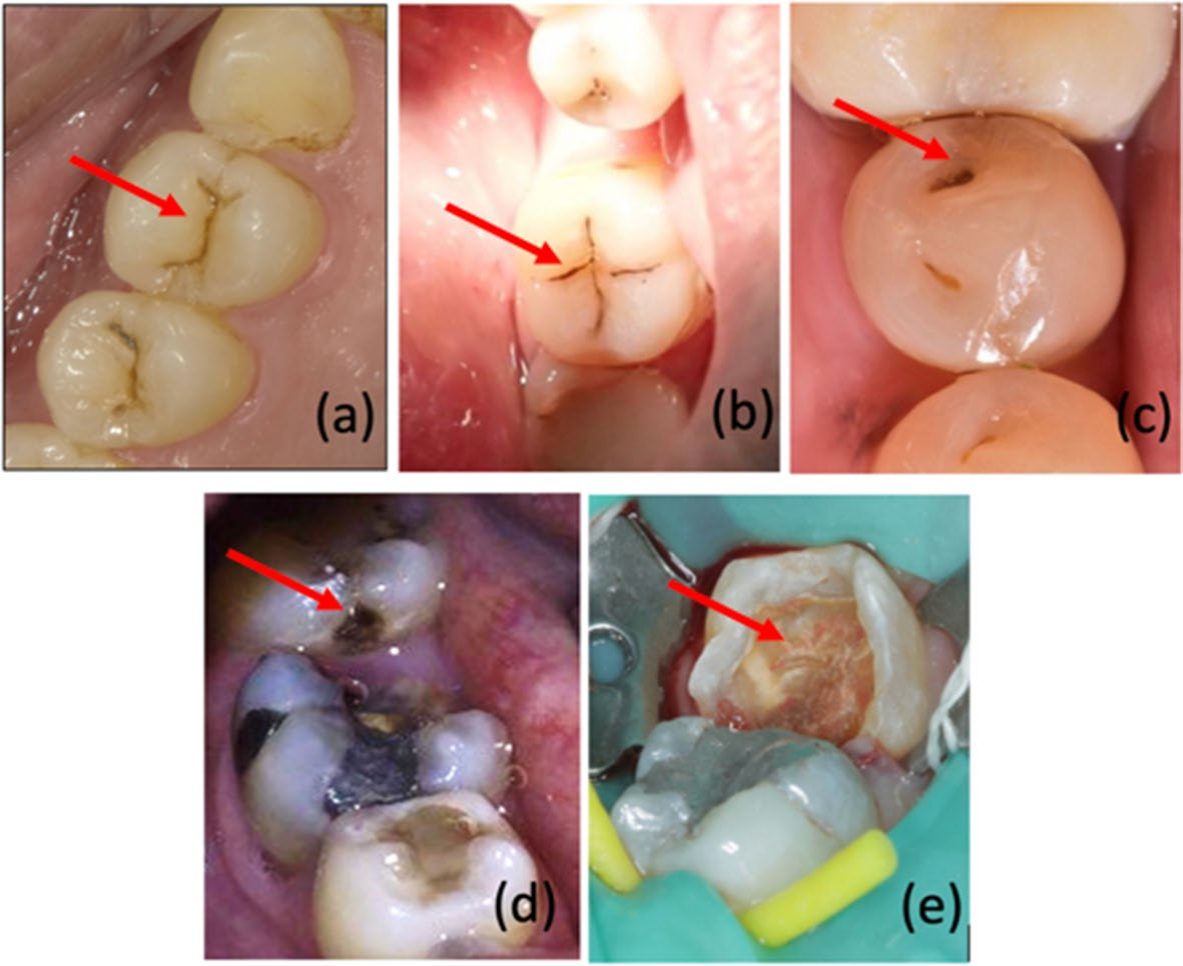
Tooth surfaces with ICDAS scores **a** OA, **b** 03, **c** 04, **d** 05 and **e** 06 as indicated by red arrow

As observed, the scoring systems are important in determining the severity of demineralized lesions. However, visual assessment paired with the scoring system is still inadequately reliable and may be ambiguous at times due to the subjective nature of measurements and difference of opinion between clinicians of varying experience. Specifically, dentists face several challenges in the scoring systems. These include difficulty in differentiating early caries (which appears as whitish or brown spot on enamel surface) from fluorosis or physiological staining, which have similar presentation. Thus, ICDAS codes 1 and 2 are sometimes combined as code 0A to indicate early caries. Figure 1 shows the severity of tooth caries lesion according to the ICDAS code. The use of the Nyvad system, on the other hand, may further damage the caries lesion on the tooth surface due to the use of sharp-ended probe.

### Clinical tools

Several advanced technologies have been adopted clinically to assess demineralized lesions. These include conventional and digital radiography, quantitative light-induced fluorescence (QLF) and digital near-infrared light transillumination. Dental radiography techniques, such as bitewing radiography and panoramic radiography, are used to diagnose and monitor tooth decay and defective fillings by capturing tooth images from different directions and positions using X-rays [16–19]. The use of ionizing radiation in dental procedures, however, gives rise to safety and health concern. The placement of X-ray sensor or film in the mouth may also cause discomfort to some patients who are prone to gag reflex [17, 20–24].

QLF works based on the principle of distinct natural fluorescence absorbed and emitted by sound and demineralized enamel [22, 25–30]. Demineralized enamel usually has higher photon scattering properties when placed under light at a certain wavelength (ranging from 380 nm to 440 nm), which results in lower fluorescence and therefore appears darker (due to limited light penetration) compared to sound enamel. Fluorescence radiance increases linearly with the amount of mineral loss and demineralization period [25, 30]. QLF is a non-destructive technique which has been used *in vitro* and *in situ* to detect and monitor progression of demineralization and remineralization longitudinally by examining changes in tooth discolouration [25]. Good correlation to transverse microradiography has also been found [22].

Digital near-infrared light transillumination (NILT) is a radiation-free tool that uses invisible light at long wavelength, specifically those in the near-infrared (NIR) spectrum, to probe samples. Utilization of such light allows greater penetration into the teeth [31]. The use of NIR light has been shown to transmit easily through the enamel layer to differentiate between a caries lesion and sound enamel [32]. Its reliability in assessing caries lesions have been compared against digital radiography and laser fluorescence. However, it showed the lowest accuracy when used for visual assessment, followed by laser fluorescence and digital radiography [31, 33].

### Research tools

Many technologies are still under rigorous research before clinical adoption. They include microradiography, microscopy, spectroscopy and OCT. Microradiography is a useful method to quantify mineral density of tooth samples [34]. Mineral density can be assessed from the amount of X-ray attenuation by dental hard tissue, where mineral density of the demineralized tooth is usually lower than sound tooth [34, 35]. Tooth samples are usually cut into thin sections of less than 100 μm for imaging [34, 36]. Besides, microradiography has been used to quantify the depth of caries lesions [36, 37] and treated as a “gold standard” in validating the efficacy of new technologies in tooth assessment. The major drawback is that it is a “destructive” tool, where a longitudinal study of the same sample is not feasible, and therefore, cannot be utilized for in vivo assessment and monitoring of teeth.

Scanning electron microscopy (SEM) is a microscopic technique which focuses high-energy electron beams at the tooth surface to generate a variety of signals. Several information can be derived from the signals, including the external morphology (surface topography, roughness and porosity), chemical composition, crystalline structure (prism density), and orientation of tooth minerals. Solid tooth samples are placed into the microscope chamber and 2D image across a selected area of ~ 5 μm to 1 cm in width is generated. Sample preparation usually relies on the analysis needed; some samples are electrically insulated using conductive coating, such as carbon for elemental analysis, while some are metal coated for high-resolution imaging [38]. Previous studies have shown that SEM can visualize distinctive features in tooth wear, such as attrition, abrasion and abfraction [38–40]. Besides, SEM can also reveal the distinct morphology of caries lesions at early stages in both primary and permanent teeth [41, 42].

Meanwhile, atomic force microscopy (AFM) is an advanced technology that provides a 3D topography of the tooth’s surface with a resolution in nanometres. Topography is obtained by “tracing” the surface with a fine tip mechanical probe during scanning. Two basic modes, which are tapping and contact mode, can be used to probe the surface of sample accurately and precisely, facilitated with small repulsive force applied to the tip through piezoelectric elements [43]. The tip is connected to a cantilever that deflect with the surface contour. The deflection changes the amount of laser light reflected into the photodiode for image formation. AFM has been used to distinguish erosive enamel due to mechanical grinding and soft drinks in a longitudinal study [44]. AFM is easier to handle compared to SEM as it does not require any prior coating of the sample surface.

Confocal scanning microscopy improves the resolution and contrast over conventional micrograph by shining a laser beam onto the sample using a small spatial pinhole to block out-of-focus light. The laser is focused at a specific field depth and is raster scanned to allow the capturing of a 2D *enface* image. Three-dimensional images can be reconstructed by capturing multiple *enface* images at different depths within the tooth sample. Confocal scanning microscopy can visualize the damage on the enamel structure clearly with spatial resolution and penetration depth in the ballpark of 1 μm and 300 μm, respectively [45, 46].

Two examples of spectroscopy that are commonly used to study tooth lesions are Raman spectroscopy and impedance spectroscopy. Raman spectroscopy is mainly used for non-destructive chemical analysis of samples, with working principle based on Raman scattering of light by chemical bonds in the sample. Majority of light scattered by the sample molecules remain at the same wavelength and do not constitute useful information. Only a small portion of the light scattered at different wavelengths will provide useful information, as the wavelengths are dependent on the chemical structure of the sample, and this is known as the Raman scatter. Polarized Raman spectroscopy is a variant system whereby the polarization states of the incident and scattered photons are carefully controlled and selected. From interactions with the tooth sample, several information can be gathered, including the polarization states, enamel composition, mineral fractions and crystalline structure of phosphate group of the tooth [47, 48]. Raman spectroscopy can be integrated with other tools, such as AFM and OCT, to attain both chemical and physical properties of samples [49, 50]. Impedance spectroscopy, meanwhile, uses electrical power to measure sample impedance, where the hydroxyapatite in the normal tooth microstructure will result in high impedance. Demineralized tooth has lower impedance due to increased porosity, in which the pores are filled with conductive fluid [51, 52]. Electrical impedance spectroscopy has been observed to show high specificity, but low sensitivity in detecting occlusal caries in permanent molars [18, 53]. A drawback in Raman and impedance spectroscopy is the bulky equipment, which are not practical for in vivo clinical use.

Profilometry, nanohardness and microhardness tests have been used to assess tooth samples and validate the aforementioned imaging modalities. Profilometry uses light or laser beam to extract the sample’s topography, such as surface roughness, which usually increases after demineralization [2, 54] and therefore has been used to diagnose eroded enamel caused by soft drinks [55]. There are two types of profilometry, depending on the probe for sample surface scanning—contact profilometry uses a metallic probe, whereas non-contact profilometry uses light or laser beam [56].

Microhardness tests are predominantly used to quantify the mechanical properties of tooth samples. Two options are available to test the microhardness, i.e. the Vickers and Knoop tests [35, 57]. Vickers microhardness test uses a symmetrical diamond-shaped indenter to indent the sample surface. The indentation (width and height) is then converted into microhardness values (HV). The Knoop microhardness test, although similar to Vickers, uses a non-symmetrical elongated diamond-shaped indenter instead and presents the measurement as the Knoop hardness number (KHN). Knoop microhardness test has been found to be more sensitive than Vickers, besides being more suitable for brittle material, such as tooth surface, because it does not penetrate deeply into the sample.

Tooth samples can also be tested in terms of nanohardness [58, 59]. Nanohardness system provides a few choices of indenter tips, such as a three-sided pyramid (Berkovich tip), spherical and cylindrical flat tips. With its small indenter, this test is suitable for heterogeneous, brittle, thin and small samples [60]. However, nanoindentation is very sensitive and the results may be influenced by subtle vibrations. The samples also have to be smoothened beforehand to provide accurate reading of the indentation size and nanohardness value.

Comparing against the aforementioned tools and imaging modalities, OCT offers the greatest advantage in terms of in vivo real-time imaging in three dimensions with short acquisition time. Its penetration depth is deeper (1–1.5 mm) compared to the confocal microscopy (0.3 mm). OCT is able to resolve the presence of both buccal and occlusal caries lesion due to its high resolution (~ 10 μm) without the use of ionizing radiation [61–63]. The system uses NIR light to probe dental tissue and the technique is non-destructive, therefore allowing longitudinal study and repeated measurements, which are important for monitoring the progression of tooth demineralization in patients. OCT scans basically do not require sample preparation, i.e. no special coating is needed as in SEM, therefore saving time, and is convenient to use. Table 4 summarizes the various modalities for assessing tooth demineralization. In the proceeding sections of this review, the role of OCT in assessing demineralization of teeth is reviewed in detail.

**Table 4:** Characteristics of the clinical and research tools for tooth assessment (Adapted from [18, 22, 35, 37, 44, 49, 51, 64–74])

	Involvement of ionizing radiation	Image dimension	In vivo*/*ex vivo	Spatial resolution	Penetration depth	Properties assessed
Transverse microradiography	Yes	2D	Ex vivo	0.3 μm–10 μm	50 μm–100 μm	Mineral density, depth
Scanning electron microscopy	No	2D	Ex vivo	0.05 μm -0.1 μm	1 μm–5 μm	Surface topography
Atomic force microscopy	No	3D	Ex vivo	Lateral: 0.001 μm Axial: 0.0001 μm	10 μm–20 μm	Surface topography
Confocal microscopy	No	3D	In vivo*, *ex vivo	Lateral: 0.18 μm–0.3 μm Axial: 0.5 μm–0.8 μm	300 μm	Surface roughness
Raman spectroscopy	Yes	2D	Ex vivo	0.2 μm–1.0 μm	0.002 μm–2 μm	Degree of crystallinity, mineral composition
Impedance spectroscopy	No	1D	Ex vivo	–	100 μm–200 μm	Porosity
Profilometry	No	2D	Ex vivo	5.0 μm	0.05 μm–1 μm	Surface topography
Microhardness, nanohardness test	No	1D	Ex vivo	–	500 μm–2000 μm	Microhardness, nanohardness value
Optical coherence tomography	No	3D	In vivo*, *ex vivo	Transverse: 25 μm﻿^a^ Axial (water/air): 12 μm /9 μm^a^	1000 μm–1500 μm	Enamel depth, optical attenuation, 3D morphology

^a^Resolution depends on the type of laser source and optics used in the system.

## The role of OCT in assessing tooth demineralization

### General system design

**Fig. 2:**
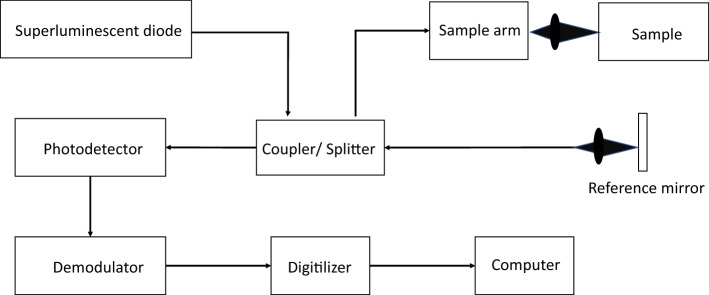
Schematic diagram of a general OCT system based on Michelson interferometer

OCT system works by the principle of low coherence interferometry. Conventional time-domain OCT system comprises a light source (e.g. superluminescent diodes and supercontinuum lasers) which emits low coherence light in the NIR range, a fibre-optic splitter and optical fibres, a photodetector with associated electronics and a computer [75]. During imaging, the light is conveyed by the optical fibre to the fibre-optic splitter, where it is split into two parts. One part is directed to the reference arm that contains a mirror, while the other part is directed to the sample arm which holds the specimen (Fig. 2). The light incident on the mirror is reflected back, whereas the light incident on the sample undergoes attenuation, scattering and reflection, with a small portion being backscattered and returned to the system. The returning light from these two arms is recombined and interfered at the fibre-optic splitter. The interference pattern is then used to form a depth scan (known as A-line) of the specimen [76]. Raster scanning of light beam across the specimen surface by a galvanometer in the sample arm can be used to generate real-time cross-sectional images of the tooth in three dimensions.

OCT measures light scattering properties of the specimen from the interference signals, which varies with composition and reflective indices of the sample’s components. The interference signals peak when the length of the reference arm matches the distance to the backscattering interface in the sample arm. To achieve precise position of the backscattering interface, a broadband light source with broad spectral bandwidth is usually adopted [76]. The resolution of the system is in the ballpark of 10–20 μm, with very high subcellular resolution of 1–5 μm achievable by hybridizing with high-numerical aperture or using state-of-the-art ultrabroadband width femtosecond Ti:sapphire laser, or Q-switch-pumped supercontinuum (QS-SC) source at the expense of field depth. The axial resolution and lateral resolution of OCT systems are decoupled. The former is influenced by the coherence gate and bandwidth of the light source, whereas the latter is affected by the numerical aperture of focusing optics (i.e. focus spot dimension). Both resolutions are important to ensure good image quality and detail [77].

The range of NIR wavelength adopted for optical imaging of dental hard tissue usually varies from 1300 nm to 1550 nm to lessen the light’s absorption for greater imaging depth [37, 74, 77–81]. The scattering of light decreases from visible to NIR region in teeth enamel because of its inorganic composition [82]. The imaging depth varied with type of tissue: 3 mm for hard tissue and 1.5 mm for soft tissue. The depth dimension in OCT images is normally displayed as a function of optical distance. Therefore corrections is usually performed by dividing the depth distance with the refractive index of the sample to obtain the true physical distance. Refractive index of enamel and dentine of human teeth, and bovine teeth are commonly taken as 1.62 and 1.50, respectively [77, 78, 83].

### Development and adaptation of OCT handpiece in dentistry

**Fig. 3:**
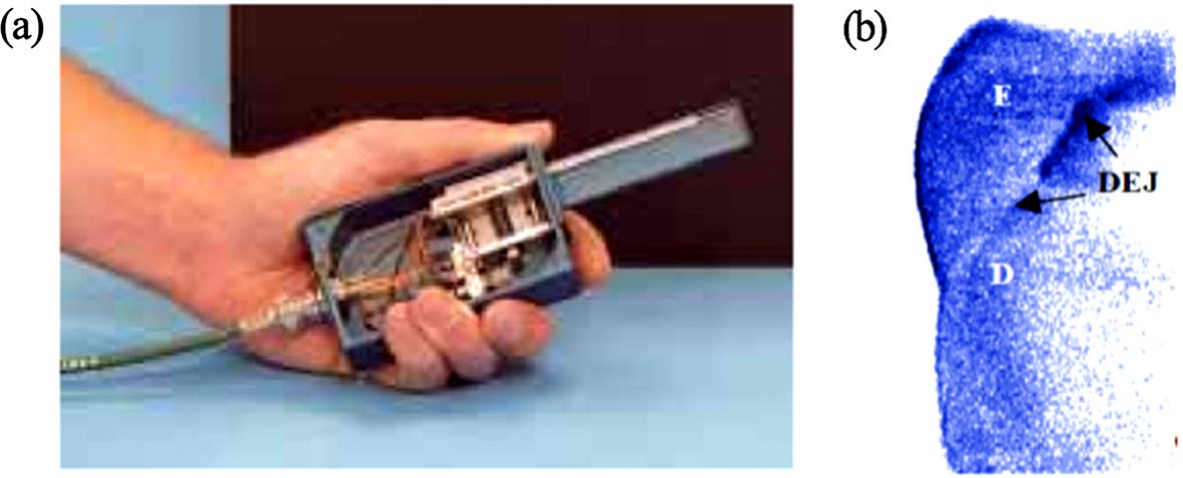
**a** The first OCT handpiece and **b **the image for visualization of enamel (**E**), dentine (**D**), and dentine–enamel junction (DEJ) by Colston et al. (Adopted with permission from [78])

OCT has been used widely in dentistry for visualizing differences in the optical properties of a tooth, such as presence of caries lesion, erosive lesion and restoration defects [84]. Colston et al. [78] first demonstrated the ability of OCT in diagnosing caries, periodontal disease and caries beneath dental restorations in 1998. The researchers performed the first in vivo study on human dental tissue using their own in-house constructed OCT system with a portable handpiece. The system was able to show significant structural details of both soft and hard dental tissues, and the dentine–enamel junction (Fig. 3), but less reliable in visualizing recurrent caries that had been treated with restoration material, such as crown and fillings [79]**.**

In 2000, Feldchtein et al. [80] performed an in vivo imaging on soft and hard tissue in the oral cavity using OCT. They used wavelengths of 830 nm and 1280 nm to achieve higher resolutions of 13 μm and 17 μm, respectively, for visualizing dental hard tissue. A special L-shaped probe had been constructed to examine the intraoral condition. Composite resin of the dental restoration was recorded by taking a series of 100 parallel B-scans, which were then 3D rendered. Secondary caries lesions between the tooth and restorative material have been demonstrated with superior structural detail and quality compared to radiography. Diagnosis of broad concave erosive lesions due to repetitive acidic challenge was characterized by the reduced mineralization and narrowing of space between enamel prisms. Restorative material showed greater light scattering compared to healthy dental hard tissues, and the type of material used for restoration may be differentiated by the differences in absorption coefficient [80].

**Fig. 4:**
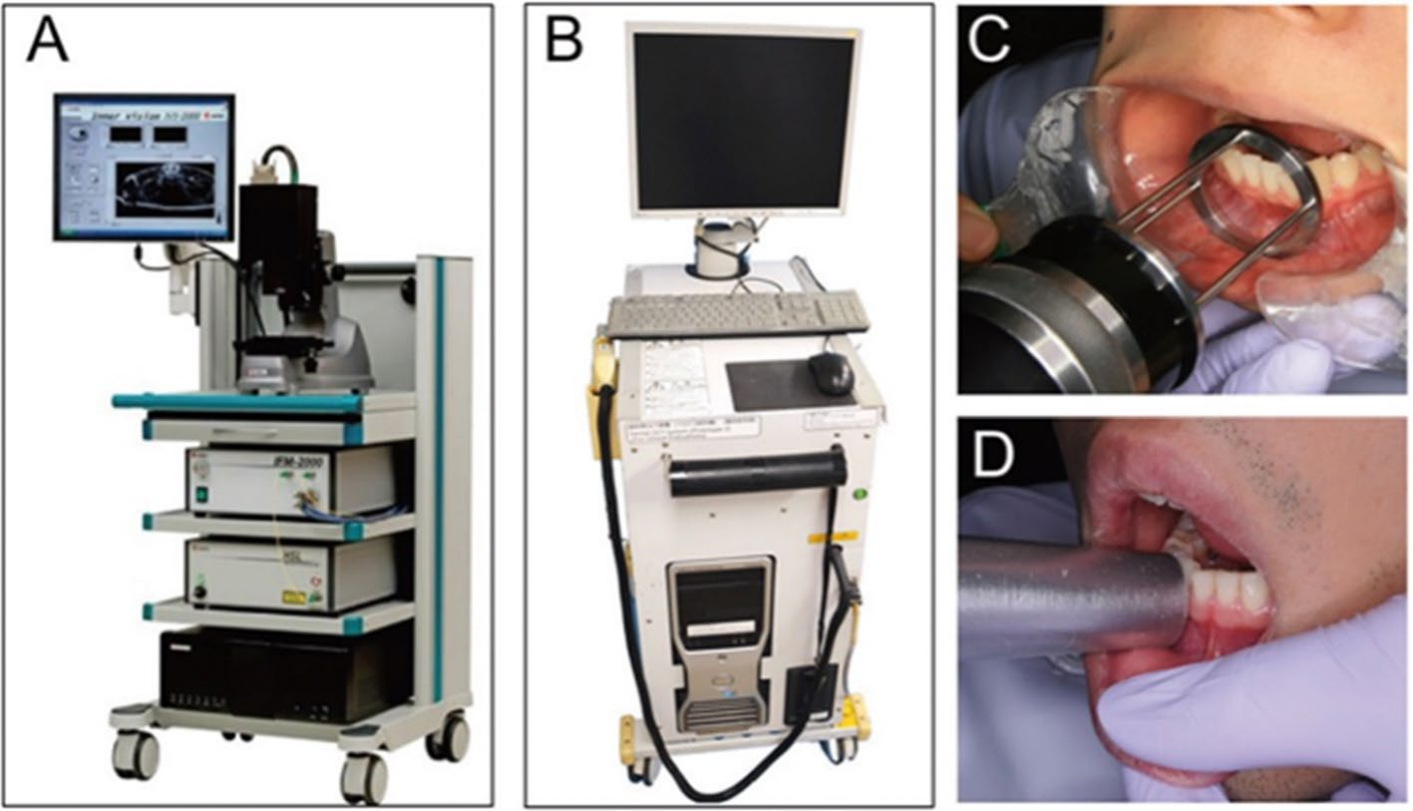
**A** Santec IV-2000 OCT system and **B** Prototype 2. The handheld probes of the IV-2000 **C** and Prototype 2 **D** systems with guides to regulate the distance from the subjects. (Adapted with permission from [86])

Recent innovations have tremendously improved the OCT handheld probe in terms of design, specifications and capability. Since the past decade, few designs have been tested in real-time in vivo clinical setting. These include the handheld scanning probe in Santec IV-2000 OCT system by Santec Corporation and Prototype 2 by Panasonic Health Care (Fig. 4) which have been demonstrated for intraoral assessment of caries [85], subgingival calculus and root cementum [86]. The systems are capable of providing impressive cross-sectional images of tooth in vivo, but the probe head design was still quite heavy and bulky, therefore not easy to manoeuvre for scanning intraorally, limiting the intraoral locations that could be accessed.

**Fig. 5:**
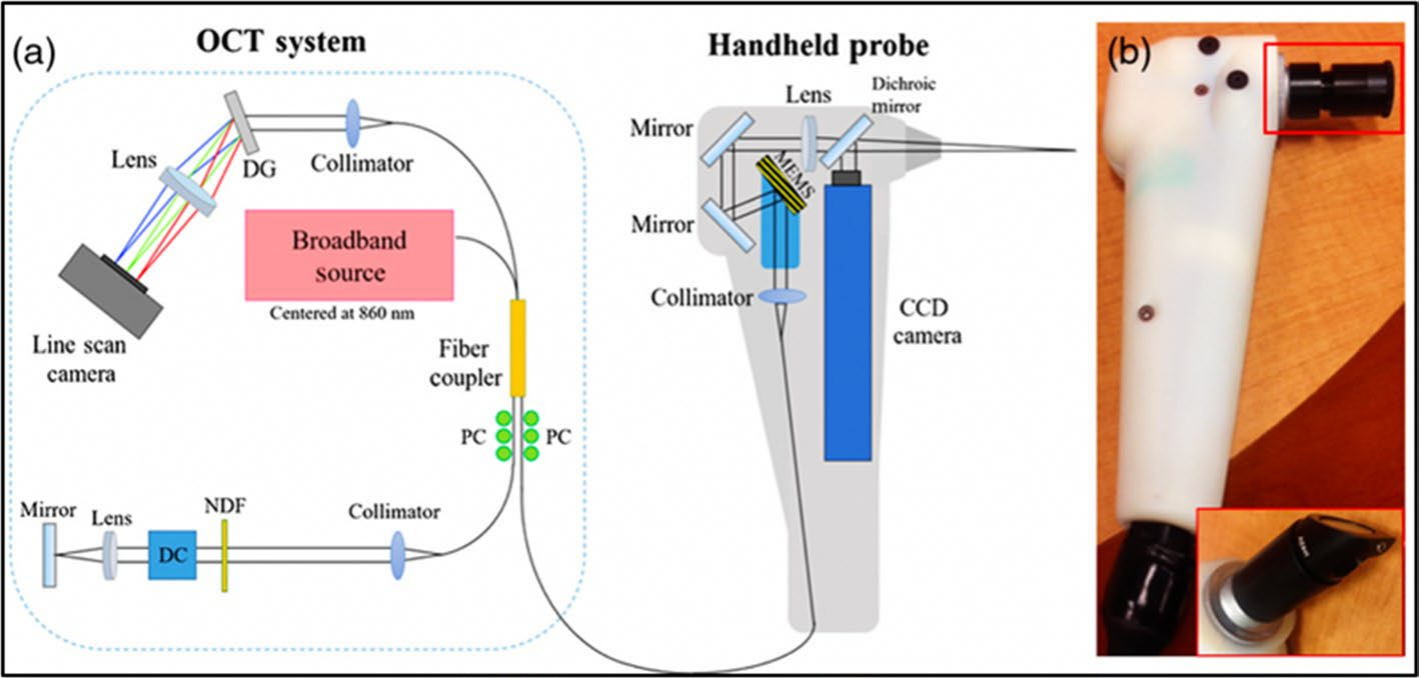
**a** Schematic diagram of the handheld OCT system probe designed by Won et al. **b** the handheld probe of the OCT system, which consists of a charge-coupled device (CCD), dispersion compensator (DC), diffraction grating (DG), neutral density filter (NDF) and polarization controller (PC). (Adopted with permission from [87])

In 2020, Won et al. have designed a more compact and ergonomic handheld spectral-domain OCT system to diagnose dental plaque and gingival health (Fig. 5) [87]. The system has a better axial and transverse resolution (~ 7 μm and ~ 25 μm in air), is capable of faster scan rate of 32 kHz and has high signal-to-noise ratio of  ~ 100 dB. One of the significant improvements on this system is the ability to modify the NIR central wavelength to suit its utilization on different dental tissues. In addition, probe–subject interface could be interchanged to enable not only the frontal imaging of anterior teeth but also right-angle imaging of posterior and lingual sides of teeth. Polarization has also been added to improve image quality and reduce artefacts.

**Fig. 6:**
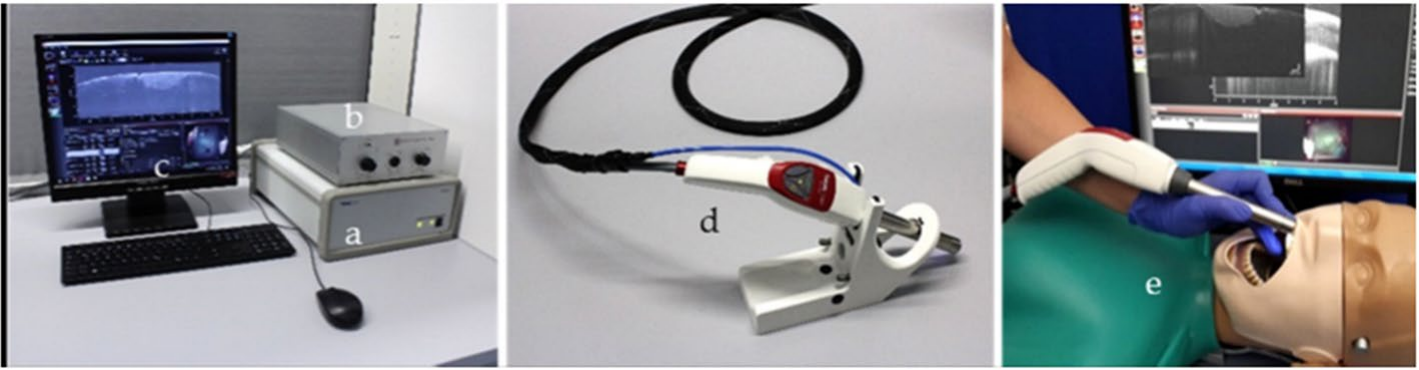
OCT system of Thorlabs GmbH **a** with custom‐designed reference arm **b**, peripheral equipment of the PC **c**, and the hand‐held intraoral OCT application probe **d** to perform test measurements on artificial sets of teeth on a patient equivalent simulation **e**. (Adopted with permission from [88])

In 2022, Medical Laser Center Lübeck (MLL) and the Institute of Biomedical Optics at the University of Lübeck published a handheld intraoral OCT application probe, which can be connected to a commercially available OCT system of the Thorlabs GmbH [88]. The OCT handheld scanner is equipped with a rigid 90º-optics endoscope (Fig. 6) for the non-invasive imaging of healthy and carious hard tooth tissues, gingiva and tooth-coloured restorations. The system was modified with a footswitch for starting and stopping 2D and 3D recording.

### Variants of OCT systems

OCT technology has evolved over the years to fulfil the clinical requirements and research purposes. Due to slow scanning rate, the first generation time-domain OCT (TD-OCT) has been surpassed by swept-source OCT (SS-OCT), spectral-domain OCT (SD-OCT) and polarization sensitive OCT (PS-OCT). These newer variants of OCT are capable of scanning with higher frame rates and shorter acquisition time [89]. SS-OCT and SD-OCT are among the favourite for dental lesion assessment.

TD-OCT depends on the mechanical motion of the reference mirror to produce intensity modulations on the photodetector to form a depth scan, which is slow [90]. SS-OCT improved over this by utilizing a fast frequency sweeping laser source coupled with a stationary reference mirror to achieve a much faster depth scan rate. The laser wavelength is rapidly swept across different frequencies to create spectrally resolved interference in the depth scan. SD-OCT, on the other hand, incorporates a broadband light source with a high-speed spectrometer to provide depth profiles, where were fast Fourier transformed and assembled into cross-sectional images. SS-OCT and SD-OCT of Thorlabs GmbH (https://www.thorlabs.com) are capable of A-scan rate as high as 60 to 200 kHz, imaging depth of 3.5 mm to 7 mm and spatial resolution of 5.5 µm to 11 µm in air. These systems provides higher SNR, accuracy and sensitivity in the detection of enamel demineralization compared with conventional visual-tactile examination [81, 85].

**Fig. 7:**
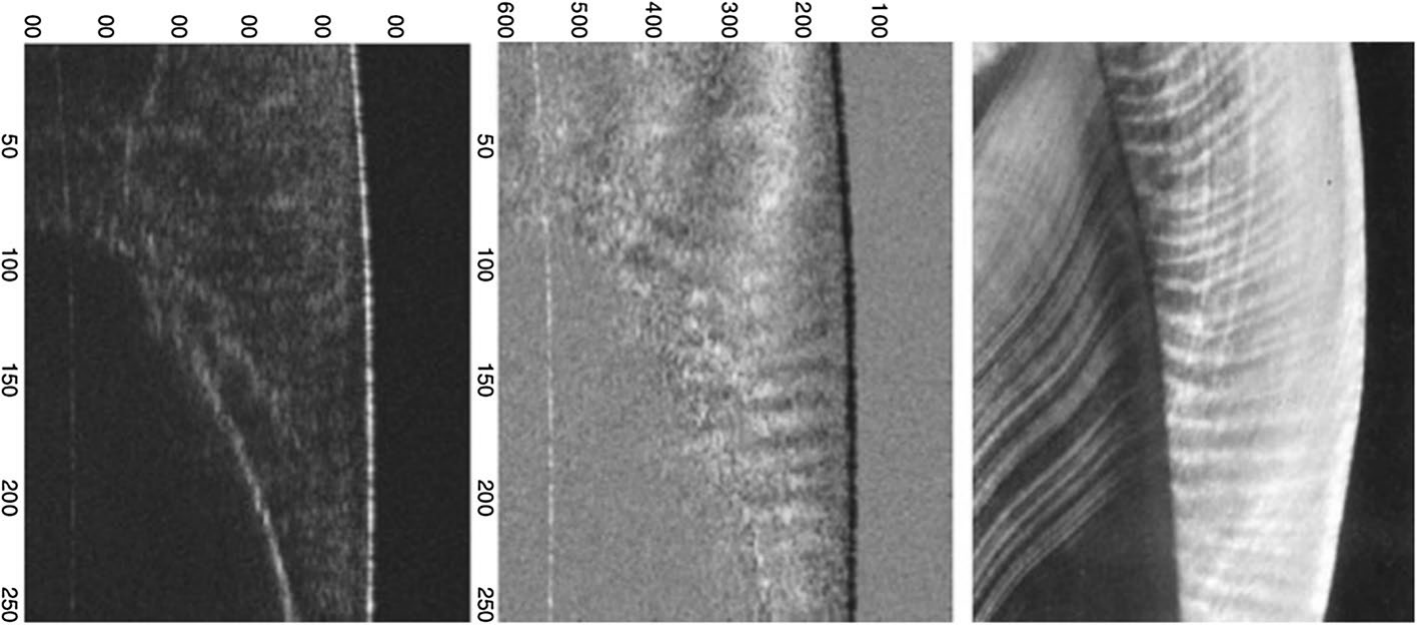
Conventional (left), PS-OCT (middle) and a ground section (right) of a human tooth reveal the superior capacity of PS-OCT to image the orientation of the enamel rods and the interrod region. (Adopted with permission from [93])

**Fig. 8:**
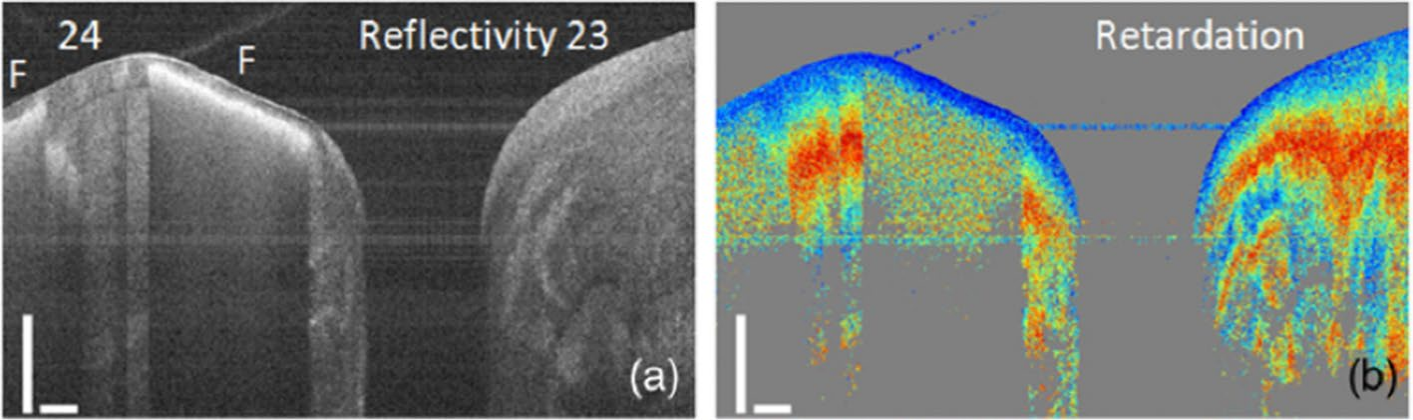
The near-surface mineralization defect (“F”) shows a strong backscattering signal in reflectivity image **a** and a high depolarization underneath in the phase retardation image **b** obtained using a PS-OCT system. (Adapted with permission from [63])

PS-OCT adds polarization contrast to standard OCT techniques. Apart from measuring the intensity of backscattered light, PS-OCT measures the polarization state of the backscattered light, which represents another form of intrinsic tissue contrast. The sample is probed with linearly polarized light and reflectivity of the sample is calculated from two orthogonal axes of polarization [63, 77, 91]. Polarization sensitivity is normally accomplished by adding a polarizing module (containing a polarizing beam splitter) to the swept-source or spectral-domain OCT system to provide additional quantitative measurements related to the polarization properties of samples, such as mineralization level, birefringence, phase retardation and backscattering [63, 90, 92]. Birefringence is an optical property associated with the orderliness of the underlying crystalline structure of minerals or microstructure of the biological sample (such as tooth in Fig. 7) that is made up of regular arrays of collagen fibres [62, 78]. Phase retardation is calculated from the cumulative Jones matrix, and it accumulates the round trip polarization effect. It has been shown that PS-OCT is able to resolve any changes in mineral density and mineral volume loss, especially in the demineralized enamel layer where the pores are highly scattered, causing depolarization of the incident light (Fig. 8) [63, 77]. Besides, PS-OCT system is also able to distinguish between occlusal and interproximal caries**,** decay under composite fillings, early root caries and both buccal and occlusal surfaces of sound and caries enamel in vivo.

Apart from those aforementioned OCT systems, other variants of OCT such as spectroscopic OCT [47, 50, 94], phase-sensitive OCT [63], multimodal OCT [109] and miniaturized OCT [95–97] have not been applied for dental demineralization assessment and may be investigated for such purpose in future studies.

### Assessment of tooth demineralization using OCT

Visual assessment is commonly carried out to identify tooth demineralization in OCT images. This assessment usually requires the involvement of few clinical practitioners to reach a consensus when ambiguous images are presented. Scoring systems, such as ICDAS and Nyvad criteria, are used in conjunction with OCT images to determine the severity of the lesion. The accuracy of caries assessment is dependent on assessor’s experience. Nakagawa et al. showed a lower inter-observer variability among group of assessors with longer experience [81].

**Fig. 9:**
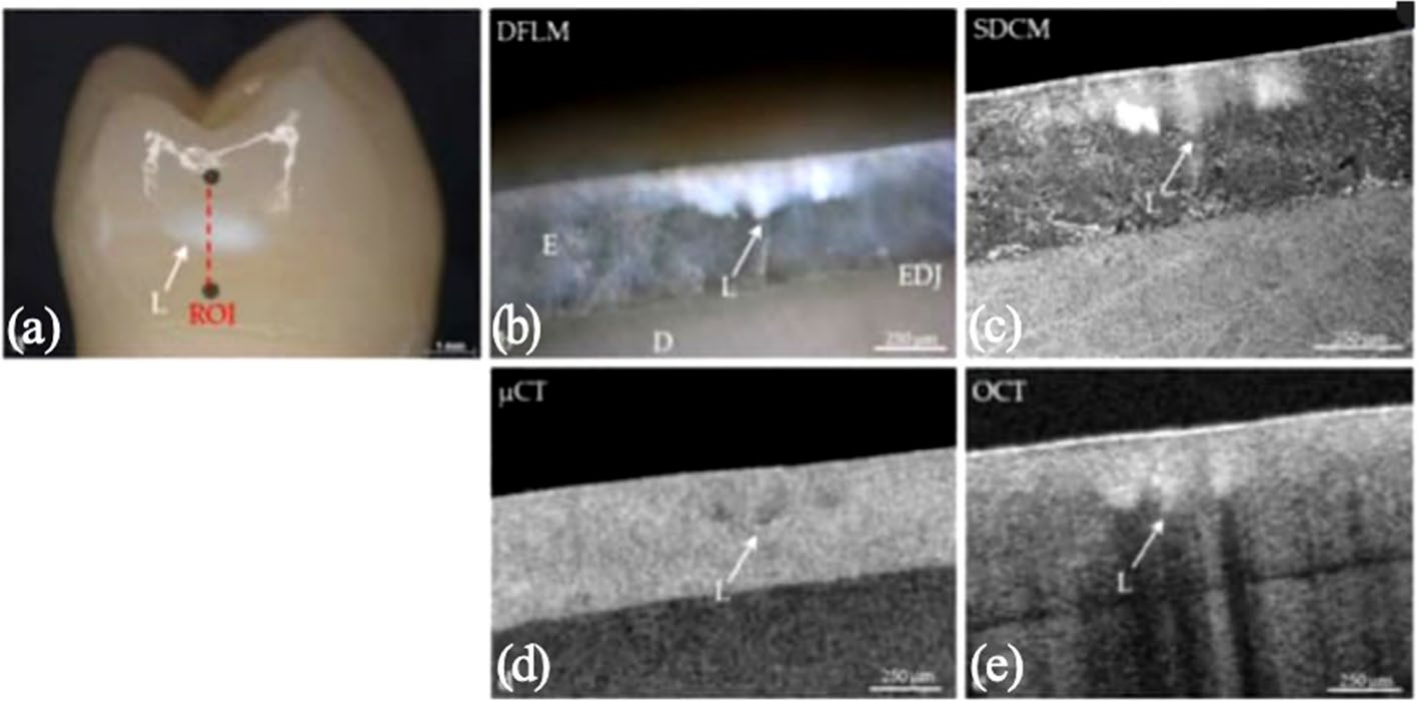
**a** A premolar tooth with caries lesion (whitish spot labelled L). Different appearances of the caries lesion under **b** dark-field light microscopy, **c** spinning-disc confocal microscopy, **d** microtomography and **e** optical coherence tomography. E: enamel, D: dentine, EDJ: enamel–dentine junction. (Adapted with permission from [98])

Caries lesions can be identified as subsurface regions of increased signal intensity in OCT images (Fig. 9) [98]. The intensity of light scattering usually increases two to three times compared to adjacent healthy region due to increased porosity from mineral loss [82]. The abundance of micro-interfaces of the porous areas in hard tissue scatters more light, consistent with caries areas shown by µCT, dark-field light microscope and confocal scanning microscopy [3].

**Fig. 10:**
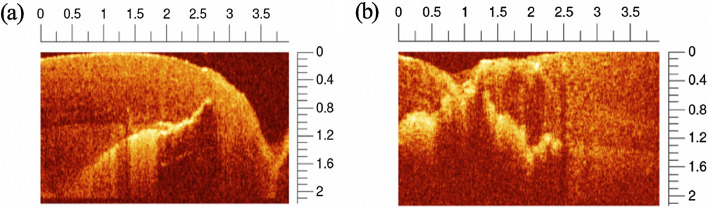
**a** B-scans of a caries lesion hidden in dentin below a composite resin prosthesis on the occlusal surface, **b** B-scan shows the extent of caries lesion underneath a small surface cavitation. (Adapted with permission from [80])

As shown in Fig. 10 (a), the optical sectioning capability of OCT plays an important role to distinguish caries lesion that is completely hidden and cannot be visually assessed from the tooth surface. This often occurs with secondary caries lesion which develops at the interface between restorative fillings and tooth tissue. Figure 10 (b), on the other hand, depicts the extent of caries lesion underneath a small surface cavitation.

There are few quantitative information that can be extracted from the OCT images to assess the caries lesion. Lesion depth and integrated reflectivity over lesion depth are good indications of the severity of demineralization [3, 37, 61, 74, 99–101]. Chan et al. [100] identified the optical lesion depth in the cross-polarization OCT image as the distance from the surface at which the reflectivity falls by 1/e^2^ from the peak value. Physical depth measurement relies on accurate knowledge of the refractive index, and the refractive index of sound tooth is 1.63 but reduces with mineral loss when demineralization occurs. Chan et al. [100] therefore have adjusted the optical depth by multiplying it with 1.55 and subtracting by 66.2 to obtain physical lesion depth, and such adjustment was based on the linear relation with in vitro lesion depth measurements in serially sectioned tooth using polarized light microscopy. Integrated reflectivity over the lesion depth (or area under the curve) has also been used by Cara et al. [101] to quantify incipient caries in human dental enamels, which showed a linear relationship with the microhardness for quantitative assessment of mineral loss in human teeth. These metrics may be valuable for monitoring of lesion progression and for assessing the effectiveness of various anti-caries agents.

**Fig. 11:**
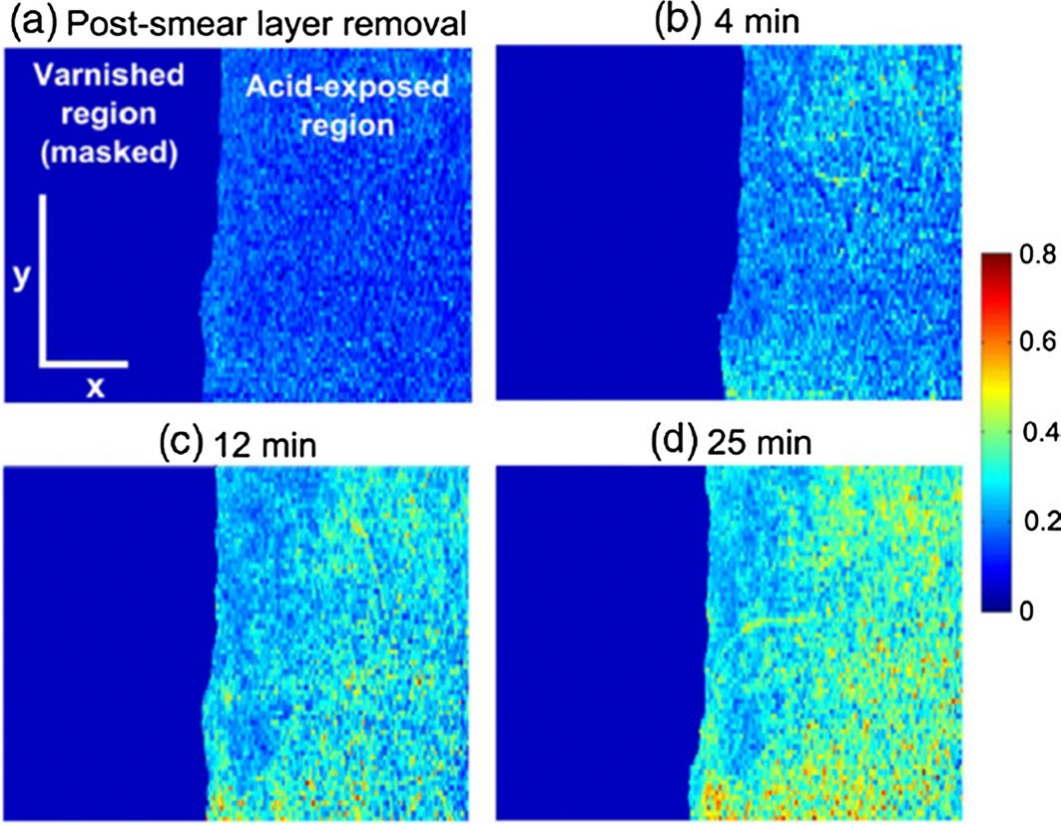
En face attenuation coefficient map at different erosion time points. Red indicates greater extent of scattering, i.e. greater extent of erosion. Colour bar represents normalized attenuation coefficient value. (Adopted with permission from [103])

In addition, optical attenuation coefficient represents light reflectivity loss with depth, and the magnitude is associated with the mineral content in tooth. The reflectivity of demineralized teeth is different from sound teeth, and the percentage of reflectivity loss increases with the duration of demineralization [102]. Extraction of attenuation coefficient is usually obtained by performing curve fitting on average A-scans using the Beer-Lambert equation and least-square method. Mandurah et al. found that the attenuation coefficient is a useful metric to monitor changes of enamel lesions during remineralization [74, 103, 104]. Madiha et al. generated *en face* attenuation coefficient maps to examine regional variation of mineral loss with different durations of erosion (Fig. 11) [103]. This metric was found adequate to distinguish between sound and demineralized tooth with high sensitivity and specificity (> 0.9).

**Fig. 12:**
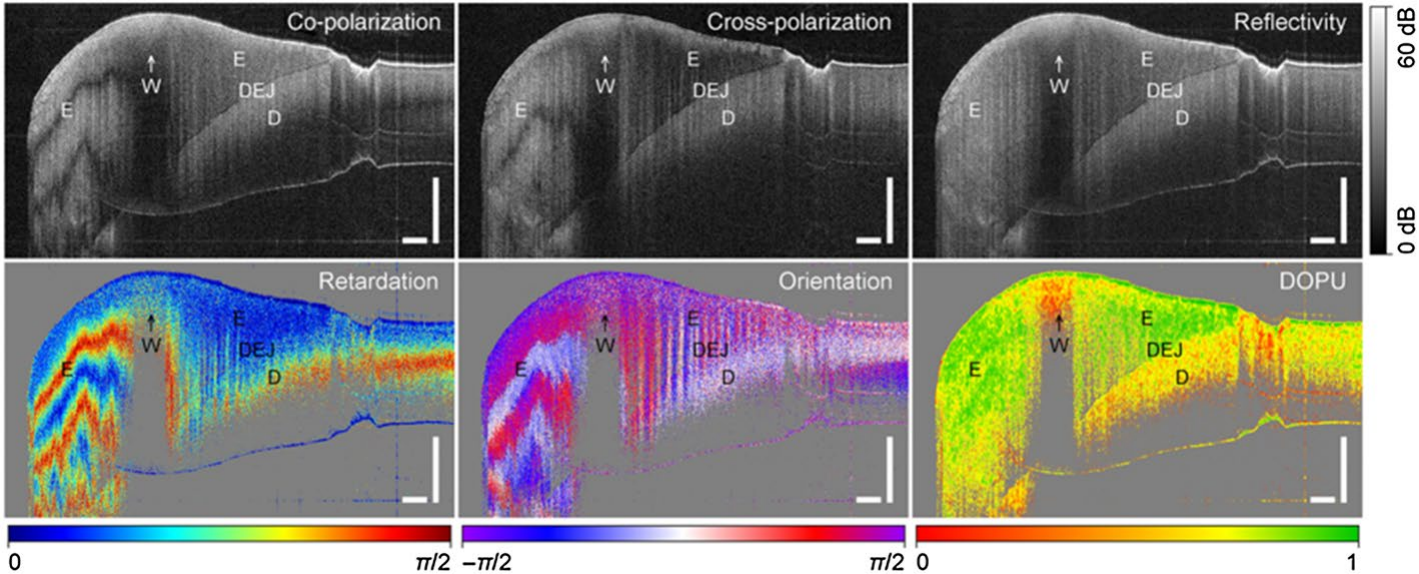
(Top row) Co-, cross-polarization and the determined reflectivity OCT B-scan showing enamel (E), the dentin–enamel junction (DEJ), dentin (D), and initial demineralization or white spot (W) of an extracted human molar tooth. (Bottom row) For polarization contrast imaging, the retardation, the fast axis orientation and the DOPU (3 × 3 kernel) are displayed. Scale bars represent 500 μm. (Adopted with permission from [105])

Depolarizing imaging using PS-OCT has also been introduced in recent years to detect proximal caries lesions based on depolarization contrast of demineralized tissue [63, 77, 91, 105]. The degree of polarization uniformity (DOPU) algorithm provides additional tissue-specific contrast and image representation on top of the normal co- and cross-polarization, reflectivity, phase retardation and fast axis orientation images. DOPU is computed as the average of Stokes vector elements and can clearly show the local variations in the polarization state of tissue [63, 77, 91]. Demineralized tissue, such as caries lesions, was recognized as regions with high depolarization in DOPU image. Golde et al. tested the algorithm on molar tooth with initial demineralization (white spot in Fig. 12), discoloured demineralization (brown spot) and advanced caries lesions, and showed that DOPU is able to differentiate early and advanced stages of carious lesions from sound dental tissue [105].

Deep learning has recently emerged as a new method for image-based caries detection. The detection mainly focused on full image classification or grading using various convolutional neural network architectures [106–110]. Specifically, Huang et al. tested AlexNet, VGG-16, ResNet-152, Xception and ResNext-101 to classify OCT images into no caries, superficial demineralization and dentine caries [110]. They achieved the best results on ResNet-152 with accuracy of 95.21% and sensitivity of 98.85%. Salehi et al., on the other hand, tested 7 methods to optimize CNN in classifying OCT images into caries and non-caries groups [106]. They found that Adam, Nadam and AdaMax are reliable optimizers, providing high accuracy of 95.45%–97.12% during training and 86.86%–88.73% during testing. They have also highlighted transfer learning models and finely tuned models of VGG16 and VGG19 as powerful methods for caries lesion classification and grading [107, 108].

Table 5 summarizes the clinical and research applications of OCT, as well as the measurements and techniques used for the assessment of tooth demineralization.

**Table 5:** The applications of OCT in research and clinical for the assessment of tooth demineralization

Authors	Application of OCT
Schneider, H., et al. [98]	Identified caries lesion that is completely hidden and cannot be visually assessed from the tooth surface. It appears as subsurface regions of two to three times increased signal intensity images compared to adjacent healthy region due to the increased porosity from mineral loss
Chan, K.H., et al.[100]	Quantified the optical lesion depth in the cross-polarization OCT image based on the refractive index of the tooth (refractive index of the tooth reduces due to the mineral loss)
Cara, A.C., et al. [101]	Quantified the caries lesion depth in human dental enamels from the integrated reflectivity of the demineralized tooth
Amaechi, B., et al. [102] Maia A.M. et al. [111]	Quantified the depth of demineralized tooth with the reflectivity loss and optical attenuation coefficient
Mandurah, M., et al. [74]	Monitor changes in enamel lesions during remineralization using attenuation coefficient that is extracted by performing curve fitting on average A-scans using the Beer-Lambert equation and least-square method on the OCT images
Habib, M., et al. [103]	Generated *en face* attenuation coefficient maps to examine regional variation of mineral loss with different duration of erosion
Golde, J., et al. [105]	Recognized demineralized tooth as regions with high depolarization in DOPU image
Salehi et al. [106–108] Huang et al. [110]	Utilized deep convolutional neural networks for caries detection (i.e. full image classification/grading of caries)

## Discussion

The potential use of OCT system for clinical dental assessment has been proven through numerous research, with improvement seen from time to time in terms of the design, procedure and methods. The benefits of the OCT system to patients are its ability to visualize and diagnose both caries and erosive lesions in vivo, including early lesions in enamel using safe and low energy NIR light. The images acquired by OCT system have a niche over advanced microradiography in terms of producing cross-sectional images as opposed to projection images which suffer from superimposition of structures. The imaging can be performed without destroying the tooth sample or exposing the patient to ionizing radiation as in microradiography and microcomputed tomography [112, 113]. Although images produced by OCT is of slightly lower resolution than confocal laser scanning microscopy, the system is capable of in vivo scanning and has greater penetration depth. Although the system is limited by the maximum scanning depth of about 2–3 mm, this depth range adequately covers most of the initial tooth defects for early detection in day-to-day clinical assessment, and for examining the effectiveness of intervention. This allows for chair side monitoring of early lesions as part of the clinical work flow in preventive management of demineralized lesions.

Since image formation in OCT always assume that light travel in straight line, intensity and morphological distortions of structure underneath the uneven tooth surface inadvertently occur when the probing light does not incident perpendicularly to the tooth surface but the effect is less studied. This warrant further investigation for precision imaging. Existing research showed the application of a refractive index matching medium (such as glycerine) onto the sample surface can improve the penetration depth and reduce surface scattering, enhancing the detectability of occlusal lesions in dentin underneath non-cavitated surfaces [114]. However, for intraoral examination, more consideration should be given to the safety of the media. Ingredients of index matching should be ingestible to ensure there is no after-effect on the patient when applied intraorally.

It is of great advantage if newer OCT technologies may allow deeper signal penetration beyond the current limits, especially to visualize lesions underneath the dentine at the occlusal region. The sitting of the occlusal region poses a difficulty for assessment, allowing only limited positioning and angulation of the OCT probe compared to buccal and mesial regions. The imaging depth of the system can be improvised by altering the wavelength of NIR light. Intraoral examination often involves both dental hard (teeth) and soft tissues (e.g. mucosa), which require different optimal probing wavelength but most of the build-ups of OCT system have a fixed central wavelength. This gives limited choice to the user; therefore, modification of the system to enable choices of central wavelength would be useful for wider clinical application.

Image acquisition time may ideally be shortened to within few seconds by increasing the frame rate. A shorter image acquisition time with less motion artefacts and better contrast are crucial to ensure accurate diagnosis. Image quality may also be enhanced by innovating a means to auto-adjust the object–probe distance as this influences the focus and the clarity of the region of interest. The protocol for intraoral application should also be improvised to ensure hygiene and to comply with infection control, avoiding unnecessary spread of infectious disease during the scanning, diagnosis and treatment process.

Apart from improving the system, the software for acquisition and image post-processing should also be improved. Various image processing techniques have been developed for other dental applications, such as averaged intensity difference detection algorithm for gingival sulcus [115], depth intensity profile analysis for microdamage detection after microimplants insertion [116], and intensity-based layer segmentation algorithm for the detection of enamel abrasion/wear, to name a few [117]. These algorithms may potentially be adapted for analysing demineralized tooth. Besides, deep learning research is very limited and currently only designed to classify OCT images to detect the presence of caries lesions. These algorithms may be devised to further localize (or segment) demineralized regions for targeted treatment and for treatment optimization. Apart from assessing optical properties, OCT elastography may be a step forward to understand and quantify the mechanical properties at the tooth areas affected by caries and erosive lesions. It would be a significant achievement if the caries lesion and erosive lesion could be differentiated non-invasively using new imaging or quantification technique in OCT.

Future studies should also cover all aspects of dental hard tissue assessments, including dentine incipient lesion in the root part, early erosive lesions, subsurface lesion with hypermineralized surface layer and deep cavitated lesion. OCT may also be hybridized with the other tools, such as autofluorescence imaging which has been shown useful to differentiate between healthy and hypo-mineralized tooth [118]. In addition, hybridization with polarized Raman spectroscopy may help provide additional spatial chemical analysis, such as the enamel composition and mineral fraction of the tooth samples through Raman scattering, in conjunction with structural and optical properties of tooth, such as lesion depth and optical attenuation coefficient [50, 69].

## Conclusion

OCT is an advance and non-invasive interferometric imaging modality that is using NIR light to visualize and assess tissue samples. In dentistry, OCT shows the ability to visualize tooth demineralization at early stage, and the subsequent progress of caries in vivo without the use of ionizing radiation. Apart from that, OCT may also demonstrate the morphological and optical changes in both sound and demineralized teeth almost similar to other advanced technologies, such as µCT. This additional information may be useful for clinical diagnosis and research that is related to oral health. Most research related to OCT and dentistry involved ex vivo studies with very limited in vivo studies. To increase the feasibility of in vivo studies and adoption of the system in day-to-day clinics, challenges in designing a lightweight probe, fast acquisition time, deeper penetration depth and clinical acquisition protocol—all while maintaining an optimum image quality and resolution—remain to be resolved.

## Data Availability

Not applicable**.**
